# Epidemiology and Diagnosis of Periodontal Diseases: Recent Advances and Emerging Trends

**DOI:** 10.1155/2014/953646

**Published:** 2014-08-25

**Authors:** S. Al-Mubarak, S. Ciancio, J. K. Baskaradoss

**Affiliations:** ^1^Dental Department, King Faisal Specialist Hospital & Research Centre, Riyadh, Saudi Arabia; ^2^Department of Periodontics and Endodontics, School of Dental Medicine, University at Buffalo, SUNY, Buffalo, NY, USA; ^3^Department of Dental Public Health, School of Dental Medicine, Case Western Reserve University, Cleveland, OH 44106, USA

Periodontal diseases, including gingivitis and periodontitis, are the most commonly occurring, yet very specific, infections of the oral cavity. When the infection is progressive, it changes from a reversible diagnosis of gingivitis to a less favorable, more difficult to reverse condition: periodontitis. Previously, research was focused on the microbiological aspects of the periodontitis. Currently, it has been well established that microorganisms alone are not sufficient for the initiation of periodontal diseases. Factors like host response, health behaviour, and stress, among other risk factors, play an important role in the presence of the disease.

Research during the past 50 years have markedly improved our understanding of the biological mechanisms of periodontal diseases. We believe that scientific partnerships among dedicated practitioners along with extensive international collaboration on research activity can further enhance existing knowledge in this field.

This special issue contains five review articles, from authors in different specialties in dentistry. In the paper entitled “*Common periodontal diseases of children and adolescents*,” Al-Ghutaimel et al. present the role of periodontal/gingival diseases in children and adolescents. This paper, highlights the importance of periodontal and gingival health in the overall wellbeing of a child.

In the paper “*The effect of orthodontic therapy on periodontal health: a review of the literature*,” S. Alfuriji et al. present a review on the role of periodontal health in orthodontic therapy. Knowledge about this interaction is of great importance not only for clinicians but also for researchers, as it has been shown that orthodontic forces tend to induce an inflammatory reaction in the periodontium.

The paper “*Risk factors of periodontal disease: review of the literature,*” by Y. A. AlJehani, provides an in-depth review of the current evidence on the potential roles of modifiable and nonmodifiable risk factors associated with periodontal disease. This review article also briefly describes the various risk characteristics that are related to periodontal diseases.

In the paper “*A new classification of endodontic-periodontal lesions*,” K. S. Al-Fouzan presents a new and modified method of classifying endo-perio lesions. The differential diagnosis of endodontic and periodontal diseases can sometimes be difficult, but it is of vital importance to make a correct diagnosis for providing the appropriate treatment.

The paper “*Diagnostic applications of cone-beam CT for periodontal diseases,*” by Y. A. AlJehani, describes the advantages of using cone-beam CT (CBCT) in the diagnosis of periodontal diseases. CBCT is a relatively new imaging modality that is widely used in general and specialized dentistry. This review is highly relevant since only few studies have appraised the role of CBCT in the diagnosis of periodontal diseases. The author has highlighted the role of CBCT in the diagnosing bony craters and defects, in measuring bone levels and in the visualization of the periodontal ligament space.

The published articles are not exhaustive representation of the recent advances in the field of epidemiology and diagnosis of periodontal diseases. Nonetheless, the papers provide a rich and many-faceted knowledge, that we have the pleasure of sharing with the readers. We would like to thank the authors for their excellent contributions and all the reviewers for their patience and cooperation.

*S. Al-Mubarak* *S. Al-Mubarak* *S. Ciancio* *S. Ciancio* *J. K. Baskaradoss* *J. K. Baskaradoss*

